# Mesenchymal stem cell-derived small extracellular vesicles facilitate repair of acute obstruction-induced colonic anastomosis injury by modulating early-stage inflammation in rats

**DOI:** 10.1186/s13287-025-04551-8

**Published:** 2025-08-06

**Authors:** Zhiwei Dong, Siran Zhou, Xianfeng Xia, Hon-Chi Yip, Kevin Kai-Chung Leung, Melissa Shannon Chan, Philip Wai-Yan Chiu

**Affiliations:** 1 Division of Upper Gastro-intestinal and Metabolic Surgery, Department of Surgery, Faculty of Medicine, The Chinese University of Hong Kong, Sha Tin, Hong Kong, N.T China; 2 Department of Endoscopy, State Key Laboratory of Oncology in South China, Guangdong Provincial Clinical Research Center for Cancer, Sun Yat-sen University Cancer Center, Guangzhou, China; 3 Multi-Scale Medical Robotics Center, Hong Kong Science Park, Sha Tin, N.T., Hong Kong, China; 4 Institute of Digestive Disease and State Key Laboratory of Digestive Diseases, Faculty of Medicine, The Chinese University of Hong Kong, Sha Tin, Hong Kong, N.T China

**Keywords:** Mesenchymal stem cells, Small extracellular vesicles, Colonic, Anastomosis, Anastomotic leakage, NF-κB, M1 macrophages, Th17 cells

## Abstract

**Background:**

Anastomotic leakage (AL), a major complication of colonic anastomoses, leads to prolonged hospital stays and increased mortality. Despite various interventions, AL incidence remains high at approximately 8%. Mesenchymal stem cells-derived small extracellular vesicles (MSCs-sEVs) have emerged as promising therapeutic agents because of their ability to exert immunoregulatory effects and promote tissue repair. Therefore, in this study, we investigated the role of MSC-sEVs in the healing of colonic anastomoses and elucidated the underlying mechanisms.

**Methods:**

In the preliminary experiments, male Sprague–Dawley rats were assigned to either a colonic obstruction or a sham surgery group, and the impact of acute colonic obstruction on colonic anastomosis healing was assessed, using an anastomotic complication score system. Based on observed impairment in ​healing progression, MSC-sEVs were administered topically in the subsequent experiments, and their therapeutic effects on anastomotic healing were analysed.

**Results:**

Acute bowel obstruction impaired colonic anastomosis healing on postoperative day four; however, the topical administration of MSC-sEVs significantly improved healing. This was mainly demonstrated by reduced immune cell infiltration, downregulation of pro-inflammatory pathways, and decreased expression of inflammatory factors, effectively controlling excessive inflammatory responses in early AL healing phases.

**Conclusions:**

In rats, MSC-sEVs effectively improve the healing of acute bowel obstruction-impaired colonic anastomoses during early-phase disease.

**Graphical Abstract:**

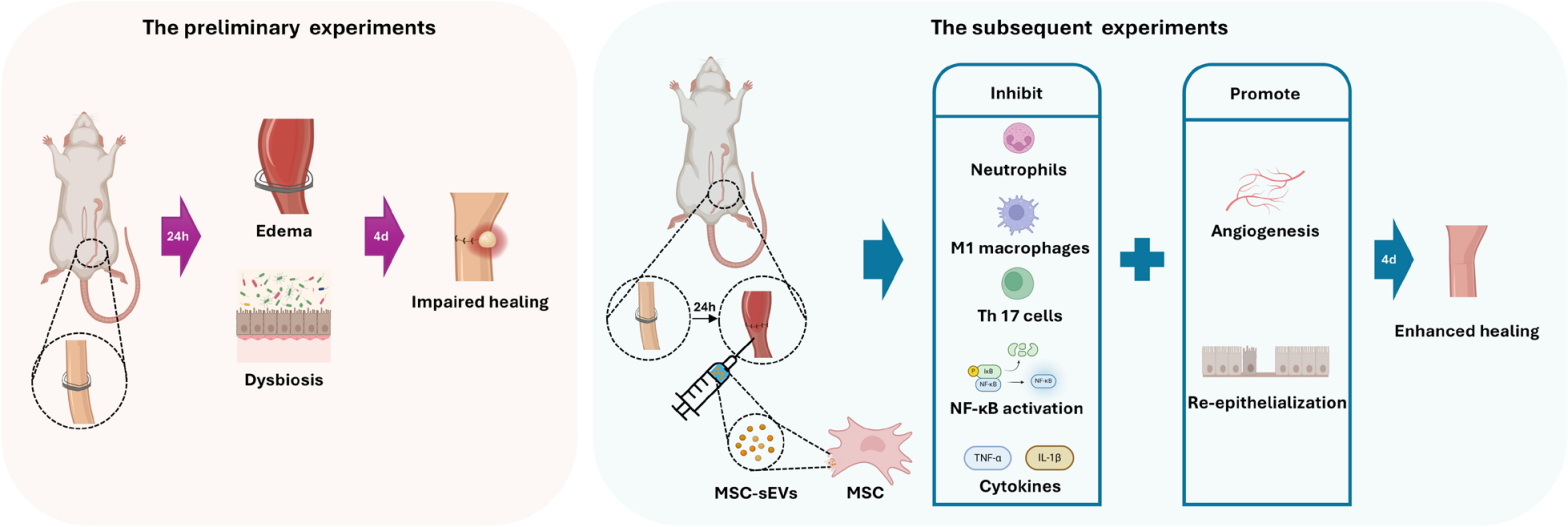

**Supplementary Information:**

The online version contains supplementary material available at 10.1186/s13287-025-04551-8.

## Background

Anastomotic leakage (AL) is a devastating complication that can occur after colonic anastomotic operations, affecting 2–19% of patients, and is associated with up to a five-fold increase in short-term mortality [1, 2]. In addition, owing to the need for additional interventions such as salvage surgery and drainage procedures, AL prolongs hospital stays and increases economic burden [3]. To date, various AL-associated risk factors have been identified, and corresponding interventions to reduce its incidence after colorectal surgery have been explored [4, 5]. However, the overall incidence of AL has remained relatively stable at approximately 8% over the past few decades, constituting a significant challenge that requires urgent attention [6]. Thus, extensive research is needed to effectively address this clinical issue.

While the exact mechanisms underlying colonic anastomoses healing remain unclear, the process can be broadly divided into three distinct phases: inflammatory, proliferative, and remodeling [7, 8]. The inflammatory phase, that occurs within the first 3–4 days after surgery, represents a critical interval during which the incidence risk of AL is highest [9]. During this phase, immune cells are mobilized, and pro-inflammatory cytokines are released, thereby contributing to the altered healing environment [7]. Following the inflammatory phase, the proliferative phase of anastomosis healing occurs within the first two weeks post-surgery, characterized by the proliferation of resident cells, including epithelial cells, fibroblasts, and endothelial cells [9]. Finally, the remodeling phase, occurring in the months after surgery, involves collagen fiber remodelling and other processes, collectively enhancing wound healing strength [10].

Due to their immunomodulatory and tissue regenerative properties, mesenchymal stem cells (MSCs) have been widely studied for the treatment of diseases related to gastrointestinal inflammation and injury [11–13]. In the context of intestinal anastomoses, MSCs contribute to the healing of anastomotic sites by reducing myeloperoxidase activity, regulating cytokine levels, and promoting angiogenesis and epithelialization [14, 15]. Studies, including our previous research, have demonstrated that MSC-derived secretome products, particularly MSCs-derived small extracellular vesicles (MSC-sEVs) or exosomes, replicate the therapeutic effects of MSCs [16–18]. Additionally, MSC-sEVs offer several advantages, including increased stability, facilitated production, and enhanced biological efficacy, and simultaneously address cell therapy-associated challenges such as immunogenicity, tumorigenic potential, and ethical concerns, thereby enhancing clinical translation [19]. However, a limited number of studies have focused on the role of MSC-sEVs in the healing of colonic anastomoses and their associated mechanisms. Consequently, in this study, we aim to investigate the therapeutic contribution of MSC-sEVs to colonic anastomotic healing and to elucidate the fundamental mechanisms involved in this process.

## Methods

### Experimental design

The work has been reported in line with the ARRIVE guidelines 2.0 and was approved by the Animal Experimentation Ethics Committee of the Chinese University of Hong Kong. Male Sprague–Dawley rats, weighing 250–350 g, were acclimatized for one week in a temperature-controlled (25–28 ℃) environment with a standard 12-h light-dark cycle prior to the experiments. The animals had access to water and a standard diet was maintained.

**Fig. 1:**
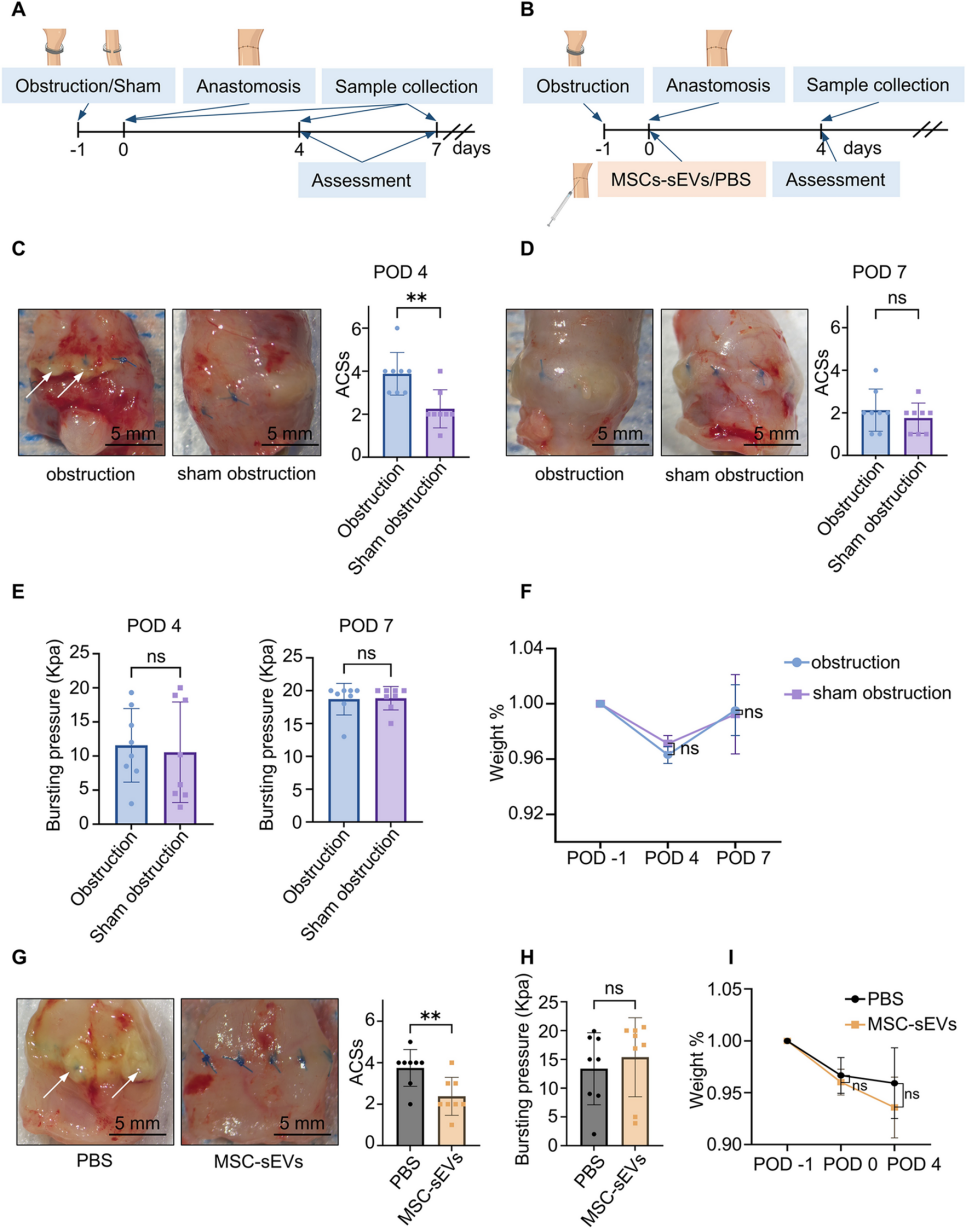
The animal experiments results. (**A**) The preliminary experiments included two groups: the obstruction and sham obstruction groups. Rats were sacrificed on postoperative day (POD) 4 or 7 (*n* = 16). (**B**) The subsequent animal experiments included two groups: the phosphate-buffered saline (PBS) group and the mesenchymal stem cell-derived small extracellular vesicles (MSCs-sEVs) group. The rats were sacrificed on POD 4 (*n* = 8). (**C**, **D**) The macroscopic assessment in preliminary experiments on POD 4 and POD 7. (**E**, **F**) The colonic anastomotic bursting pressure and weight changes in preliminary experiments. (**G**) The macroscopic assessment in subsequent experiments on POD 4. (**H**, **I**) The colonic anastomotic bursting pressure and weight changes in subsequent experiments. All data are shown as means ± SD. * * *P* < 0.01

In the preliminary experiments (Fig. 1A), 32 Sprague–Dawley rats were randomly assigned to either an obstruction group, which underwent a colon obstruction procedure, or a sham obstruction group, which underwent a corresponding sham procedure. Twenty-four hours later, both groups underwent colectomy and primary anastomosis (CPA). Half of the rats were euthanized on postoperative day (POD) 4 through an intraperitoneal injection of 1 ml of 20% pentobarbital, while the other half were euthanized on POD 7 for sample collection and macroscopic assessment using the anastomotic complication score system (ACSs) (Table S1) by a blinded observer. The sample size was determined through power analyses based on previous data.

In the subsequent experiments (Fig. 1B), 16 additional Sprague–Dawley rats were randomly assigned to either the MSC-sEVs group or the phosphate-buffered saline (PBS) group. All rats initially underwent a colon obstructive procedure, followed by CPA and a topical injection subserosally around the anastomosis of either 60 µL of MSC-sEVs concentrated solution (the total protein concentration was 1.5 µg/µL) or an equal volume of PBS after 24 h. All procedures mirrored those of the preliminary experiments. The rats were euthanized on POD 4 based on the preliminary study results. Conditions of the rats were monitored, and analgesics were administered after each operation, as well as on the following two days or as needed to alleviate pain. No animals exhibiting serious injuries or signs of severe pain or distress were euthanized prior to the designated endpoint.

### Animal model

The rats were anesthetized via intraperitoneal injection using a mixture of 10% ketamine, 2% xylazine, and sterile 0.9% sodium chloride NaCl in a 3:2:3 ratio, at a dosage of 0.5 ml per 250 g of body weight, administered 5 min prior to the surgical procedures. To induce bowel obstruction, a midline laparotomy was performed, and a medical-grade silicone ring (6.5 mm inner diameter, 3 mm width) was placed around the descending colon between two marginal blood vessels, approximately 2 cm proximal to the peritoneal reflection. The ring was closed with a 7 − 0 polypropylene suture (Fig. S1A), and the laparotomy was closed in two separate layers. In the sham procedure, the silicone ring was cut into two pieces and placed around the colon without applying pressure (Fig. S1B).

For the anastomosis [20], a midline laparotomy was performed at the same site, and the colon condition was observed (Fig. S1C D). A 15 mm segment of the descending colon, including the silicone ring, was resected, and the excised colon tissue and faecal samples were collected for further analysis. The ends of the colon were carefully trimmed to ensure adequate blood perfusion. Following disinfection, a primary end-to-end (serosa to serosa) anastomosis was performed using evenly spaced interrupted 7 − 0 polypropylene sutures (Fig. S1E). The number of sutures used in each model was recorded for statistical analysis. Additionally, the omentum between the proximal colon and spleen was dissected to prevent tension on the anastomotic site. An inflation test was conducted by clamping the proximal colon, injecting air into the colon via a blunt needle inserted into the rectum, and identifying any air leakage for repair. The sequence of operations for both groups was randomly determined.

### Anastomotic bursting pressure

All anastomotic bursting pressure tests were conducted in situ (Fig. S2). Specifically, a partial incision was made in the colon 3 cm proximal to the anastomosis, and a tube was inserted distally. The colonic anastomosis was submerged in warm saline and the tube was connected to a syringe and pressure manometer (NIDEC COMPONENTS CORPORATION, Kyoto, Japan). The colon was gradually inflated until air bubbles appeared at the anastomotic site, and the pressure was recorded immediately. To prevent tissue rupture, inflation was ceased once the pressure reached 20 KPa.

### RNA extraction, sequencing, and data analysis

Total RNA was extracted from the rat colon anastomotic tissues using the TRIzol reagent, followed by purification with a Qiagen RNeasy Mini Kit (Qiagen), according to the manufacturer’s instructions. Sequencing was performed on the Illumina NovaSeq 6000 platform (Illumina Inc.). Differential gene expression analysis was conducted using the R package DESeq2, with a fold change threshold of ≥ 2 and a *p*-value threshold of < 0.05 indicating significant changes. Gene Ontology (GO) (http://www.geneontology.org/) and Kyoto Encyclopedia of Genes and Genomes (KEGG) pathway analyses were conducted to analyze differentially expressed genes (DEGs) for biological functions and signaling pathways, respectively, with statistical significance set at a *p* < 0.05.

mRNA levels were validated using RT-qPCR. All primers were obtained from BGI Genomics (Shenzhen, China) and designed based on reference sequences from the National Center for Biotechnology Information (Table S2). RT-qPCR was performed using TB Green Premix Ex Taq (Takara, Kusatsu, Japan) on a QuantStudio 7 Real-Time PCR System (Applied Biosystems, MA, USA). Relative mRNA expression was calculated using the 2^−ΔΔCT^ method and normalized to that of GAPDH. Statistical significance between the two groups was assessed using Student’s t-test, and the *p*-value was set at 0.05.

### Faecal and colon mucosal sample collection, DNA isolation, and 16 S ribosomal RNA sequencing

Total bacterial DNA was extracted from faecal and colonic mucosal samples obtained from rats in both obstruction and sham obstruction groups. For colonic mucosal samples, the 15 mm segment of the resected colon was opened longitudinally and gently washed twice with sterile PBS to remove adhered faecal matter. The mucosa was then collected using a sterile surgical blade for total bacterial DNA extraction. Faecal samples were collected from the proximal intestinal lumen near the obstruction site, and equal quantities of these samples were used for DNA extraction. DNA extraction from both faecal and colonic mucosal samples was performed using QIAamp DNA mini kit (Qiagen, Hilden, Germany), following the manufacturer’s instructions. DNA library preparation and 16 S ribosomal RNA (rRNA) gene sequencing were conducted on an Illumina NovaSeq 6000 platform (illumine Inc., CA, USA) by NovoGene in Tianjin, China.

### Hydroxyproline measurements

A section of the colon wall, approximately 1/3 of its circumference and 0.5 cm in length, encompassing the anastomosis, was collected for hydroxyproline measurements. The hydroxyproline concentration in the colonic anastomosis was measured spectrophotometrically using a hydroxyproline assay kit (ab222941, Abcam, Cambridge, UK) as per manufacturer’s instructions. Measurements were performed at 560 nm. Data were expressed as µg of hydroxyproline per mg of tissue.

### Histological analysis

Colonic anastomotic tissues were fixed with 4% paraformaldehyde, gradually dehydrated, embedded in paraffin, and cut transversely into 5 μm-thick sections. Tissue sections were dewaxed and stained with haematoxylin and eosin and Periodic Acid-Schiff to evaluate re-epithelization and goblet cell (periodic acid Schiff-positive).

Masson’s trichrome staining was performed to assess collagen fibre accumulation in anastomotic tissue sections. Staining procedures were performed as per manufacturer’s instructions (Masson’s trichrome staining kit, Epredia, NH, USA). To assess the total collagen fibres, ten fields were randomly selected and magnified at 400 × in the anastomotic zone stained with Masson’s trichrome. The collagen volume fraction in these fields was calculated as the ratio of the blue-stained (indicative of collagen) area to the total tissue area. The mean of 10 such measurements was then taken as the final result.

### Immunohistochemistry, Immunofluorescence staining, and in situ cell death detection

Histological sections were deparaffinised and rehydrated. Antigens were retrieved via heating of the sections in antigen retrieval buffer (Abcam, Cambridge, UK) in a pressure cooker for 8 min, followed by cooling to room temperature. All the sections were incubated with 3% hydrogen peroxide at room temperature for 10 min to inhibit endogenous peroxidase activity. Subsequently, sections were incubated in a humidified chamber at room temperature for 30 min with 10% normal goat serum (containing Triton). Primary antibody (PCNA, ab152112, 1:700, Abcam), CD 31(ab28364, 1:400, Abcam), and myeloperoxidase (ab208670, 1:2000, Abcam, Cambridge, UK) incubation was performed overnight at 4 ℃. The following day, the sections were incubated with either HRP anti-mouse IgG (MP-7402, Vector Laboratories) or HRP anti-rabbit IgG (MP-7401, Vector Laboratories, CA, USA) for 1 h at room temperature. Results were visualized using the DAB reagent (Dako, Glostrup, Denmark) and the nuclei were counterstained with haematoxylin. The sections were dehydrated, cleared, and mounted using DPX.

Immunofluorescence staining was performed using a similar protocol as described previously to detect the expression of CD68 (Alexa Fluor^®^ 647, ab305214, 1:50, Abcam), iNOS (ab283655, 1:50, Abcam), CD86 (942-RBM4-P1ABX, 1:200, NeoBiotechnologies), CD206 (ab64693, 1 µg/ml, Abcam). The second antibodies used were goat anti-rabbit IgG (Alexa Fluor^®^ 488, ab150077, 1:1000, Abcam, Cambridge, UK) and goat anti-mouse IgG (Alexa Fluor^®^ 594, ab2534079, 1:1000, Invitrogen). After applying the secondary antibody, cell nuclei were counterstained and mounted using SlowFade Diamond (Invitrogen, CA, USA).

Colon tissue sections were processed in the same manner as described above and stained using a fluorescent In Situ Cell Death Detection Kit, as per manufacturer’s instructions (Roche, Basel, Switzerland).

The slides were examined under a microscope (Leica Camera, Wetzlar, Germany) and the captured images were analysed using the ImageJ software. An independent observer quantified data in a blinded manner.

### Polychromatic immunofluorescent staining

The colonic anastomotic sections were prepared as above, then were stained according to the instructions of four-color multiplex fluorescence immunohistochemical staining kit (Absin, Catalog No.abs50012). Primary antibodies involved CD3 (ab16669, 1:200, Abcam, Cambridge, UK), CD4 (#25229, 1:200, Cell Signalling Technology, MA, USA), and IL-17 A (ab318150, 1:500, Abcam, Cambridge, UK). The nuclei were stained with DAPI before sealing, all sections were captured by Pannoramic MIDI (3D HISTECH, Budapest, Hungary) and analysed using Pannoramic Viewer software (3D HISTECH).

### Isolation and characterisation of adipose-derived MSC

SD rats weighing 250–350 g were used to obtain adipose-derived MSCs. Subcutaneous white fat was harvested from the inguinal region and digested with 0.1% collagenase I at 37 °C for 30 min. After digestion, the stromal-vascular fraction was collected via centrifugation for 5 min at 300 × g. Cells were resuspended and cultured in Dulbecco’s Modified Eagle Medium (DMEM) with low glucose (Gibco, Thermo Fisher Scientific, MA, USA), supplemented with 10% fetal bovine serum (FBS; Gibco, Thermo Fisher Scientific, MA, USA), antibiotics (100 U/mL penicillin, 100 µg/mL streptomycin, and 0.25 µg/mL amphotericin B; Gibco, Thermo Fisher Scientific, MA, USA) and 200 mM GlutaMAX (Gibco, Thermo Fisher Scientific, MA, USA). Cell-culturing was performed in a 5% carbon dioxide atmosphere at 37 °C until they reached 80–90% confluency. Passage 3–4 cells were used for experiments.

The cell surface immunophenotype was assessed using flow cytometry, testing positive for CD29, CD90, CD105, CD44 and negative for CD45 according to the criteria from the International Society for Cell & Gene Therapy (ISCT^®^) [21].

For differentiation assays, cells were seeded at 1 × 10^4^ cells/cm^2^ in 12-well plates for osteogenesis and adipogenesis and 2 × 10^4^ cells per well in 96-well round-bottom plates for chondrogenesis. Lineage-specific differentiation media from the StemPro Differentiation Kit (Gibco, Thermo Fisher Scientific, MA, USA) was used. After 14 (adipogenesis and chondrogenesis) or 21 (osteogenesis) days, the cells were fixed and stained with Oil Red O, Alcian Blue, or Alizarin Red S to confirm adipogenic, chondrogenic, and osteogenic differentiation, respectively.

### MSC-sEVs extraction and characterisation

When the MSC reached 80–90% confluency, the medium was replaced with FBS-free DMEM after washing twice with PBS. The culture supernatant was collected after 48 h, and MSC-sEVs were extracted using differential centrifugation according to a standard protocol. Briefly, the supernatant was centrifuged at 2,000 × g for 10 min and 20,000 × g for 40 min to enrich the debris and larger EVs. The supernatant was collected and centrifuged at 100,000 × g for 70 min to enrich the sEVs. The pellet was resuspended in PBS and subjected to centrifugation at 100,000 × g for 70 min to further enrich the sEVs. All centrifugation steps were performed at 4 °C. After resuspending the pellet in PBS and adjusting the total protein concentration to 1.5 µg/µL, the solution was filtered through a 0.22 μm filter. The small sEVs were subsequently stored at -80 °C.

The size distribution of sEVs was analysed using a Nanoparticle Tracking Analyzer, specifically the ZetaView^®^ (PMX-120, Particle Metrix, Inning, Germany). The sEVs protein concentration was determined using a detergent-compatible protein assay kit (Bio-Rad Laboratories, CA, USA). The presence of specific protein markers in sEVs (CD63, Alix, CD81, and Tsg101) was validated using western blot analysis. The morphology of sEVs was observed using transmission electron microscopy (Hitachi H-7700, Hitachi, Japan).

### Cell lines and cell culture

The murine macrophage cell line (RAW264.7) and the human normal colon epithelial cell line (CCD 841 CoN) were purchased from the American Type Culture Collection (ATCC, Manassas, VA). RAW264.7 was cultured in the DMEM medium, and CCD 841 was cultured in the MEM medium. Both DMEM and MEM were supplemented with 10% FBS (Thermo Fisher Scientific, Waltham, MA) and Antibiotic-Antimycotic (Thermo Fisher Scientific). The RAW264.7 cultured in that medium were defined as M0 macrophages. The human umbilical vein endothelial cells (HUVECs) were provided by Fenghui Biotechnology (Hunan, China).

HUVEC was cultured in a vascular cell basal medium supplemented with an endothelial cell growth kit (Fenghui Biotechnology). All cells were incubated at 37℃ and 5% CO2, and the medium was replenished every three days. When cells reached 80–90% confluency, they were detached using 0.5% Trypsin-EDTA (Thermo Fisher Scientific) and passaged.

### Tracking MSC-sEVs with PKH26 staining in vitro and in vivo

MSC-sEVs were labelled with PKH26 (MINI26-1KT; Sigma-Aldrich, MO, USA). To halt staining, PBS containing 1% bovine serum albumin (BSA) was added. The mixture was then filtered through a 10 kDa filter unit (UFC910024), and the unfiltered portion was resuspended in PBS. The labelled sEVs were enriched via another round of ultracentrifugation and resuspended in a complete medium for further experimentation.

For the in vitro cellular uptake experiment, CCD 841 CoN cells, HUVECs, and RAW 264.7 cells were seeded in 12-well plates with sterile circular coverslips at the bottom. Once the cells reached 60–80% confluence, the complete culture medium was replaced with either PKH26-labelled MSC-sEVs or an equal volume of PBS as a control and co-cultured for 6 h. After fixing the cells with 4% paraformaldehyde, actin was labelled using ActinGreen 488 ReadyProbes Reagent (Thermo Fisher Scientific). Cell nuclei were labelled using SlowFade Diamond Antifade Mountant (Thermo Fisher Scientific, Sunnyvale, CA, USA). The cells were visualized under a fluorescence microscope (Eclipse Ts2; Nikon Corporation, Tokyo, Japan).

For in vivo tracking of MSC-sEVs, the labelled MSC-sEVs were injected sub-serosally around the colon anastomosis. The rats were sacrificed 12 h post-injection, and frozen tissue sections were subsequently examined via fluorescence microscopy, following the aforementioned methods.

### Cell migration assay

For the migration assay, 1 × 10^5^ CCD 841 CoN cells were plated in serum-free media on inserts with 8 μm pores (353097, Corning, NY, USA), and migration towards conditioned media (5% FBS-DMEM containing MSC-sEVs or PBS) was measured after 8 h. Inserts were formalin-fixed and stained with 0.1% Crystal Violet. Four images of each insert were captured at 100× magnification using a Nikon Eclipse E600 microscope (Nikon Corporation, Tokyo, Japan) and quantified using ImageJ software. All experiments were performed in triplicate.

### CCK-8 assay

A cell proliferation assay of CCD 841 CoN cells was performed using a cell counting kit-8 (CCK-8, Abcam, Cambridge, UK), as per manufacturer’s instructions. Briefly, 100 µL of cell suspension containing either MSC-sEVs or PBS was seeded in a 96-well plate (3000 cells/well). Then 10 µL of CCK-8 solution was added to each well at different time points, followed by incubation at 37 ℃ for 2 h. The number of viable cells was determined by measuring absorbance at 450 nm. All experiments were performed in triplicates.

### Co-culture of RAW 264.7 cells with MSC-sEVs

RAW 264.7 cells were seeded and cultured in a 6-well plate until reaching a confluency of 50–60%. The medium was then replaced with a complete medium supplemented with 100 ng/ml LPS (Sigma-Aldrich, MO, USA). After 6 h, the supernatant was discarded, and the cells were washed twice with PBS. Subsequently, a complete medium containing either PBS or different concentrations of MSC-sEVs (15 µg/ml or 20 µg/ml) was added for co-culture. Following the 18 h co-culture period, the supernatant was collected for enzyme-linked immunosorbent assay (ELISA) and the cells were harvested for flow cytometry or protein extraction.

### ELISA

Concentrations of TNF-α and IL-6 in the supernatant were measured using highly sensitive ELISA kits obtained from FineTest (EM0183-HS and EM0121, FineTest, Wuhan Fine Biotech Co., Ltd., Wuhan, China), as per manufacturer’s instructions. Absorbance was measured at 450 nm using a microtiter plate reader. Concentration values were expressed as pg/mg protein, as specified in the manufacturer’s guidelines.

### Protein extraction and Western blots

Colon tissues and cells were lysed in RIPA lysis buffer (50 mM Tris, 150 mM sodium chloride, 1.0% NP-40, 0.5% sodium deoxycholate, and 0.1% sodium dodecyl sulfate; pH 8.0) supplemented with a phosphatase inhibitor cocktail (Sigma-Aldrich, MO, USA) and a protease inhibitor cocktail (Sigma-Aldrich, MO, USA). The lysates were centrifuged at 16,000 × g for 20 min at 4 °C, and the supernatants was collected and stored at -80 ℃. The protein concentration of each sample was determined using a BCA protein assay kit (Thermo Fisher Scientific) as per manufacturer’s instructions. The protein lysates were then denatured by boiling at 95 °C for 15 min together with a protein loading buffer.

For western blotting, equal quantities of protein were loaded onto sodium dodecyl sulfate-polyacrylamide gel electrophoresis gels and transferred onto polyvinylidene difluoride membranes (Millipore, MA, USA). The membranes were blocked with 5% BSA in Tris-buffered saline containing 1% Tween-20 (TBST) for l h at room temperature. Primary antibodies (Table S3) were prepared, and membranes were incubated overnight at 4 °C. After three washes with TBST, the membranes were incubated with horseradish peroxidase (HRP)-conjugated secondary antibodies (diluted 1:3000, #7074, #7076; Cell Signalling Technology, MA, USA) for 1 h at room temperature. Signal detection was performed using Clarity Max Western ECL Substrate (1705062; Bio-Rad Laboratories, CA, USA).

### Flow cytometry analysis

When the cells reached 80% confluence, they were washed with PBS and incubated with 3% BSA for 1 h to block non-specific antigen binding. Cells were resuspended after centrifugation and incubated with the following fluorescein-conjugated antibodies for 30 min at 37° C in the dark: FITC anti-CD105 antibody (Abcam), FITC anti-CD90 antibody (Abcam), PE anti-CD44 antibody (Abcam), FITC anti-CD45 antibody (Abcam, Cambridge, UK), PE anti-F4/80 (BioLegend), AF488 anti-iNOS (BioLegend, CA, USA). For intracellular staining, an Intracellular Fixation & Permeabilization Buffer Set (eBioscience, CA, USA) was used as per manufacturer’s instructions. Cells were collected and washed with PBS after centrifugation. Fluorescence was evaluated using a CytoFLEX LX Flow Cytometer (Beckman Coulter, CA, USA), and further analysis was performed using FlowJo software (Tree Star Inc., OR, USA).

### Statistical analysis

Experiments were conducted in triplicate and the data are presented as mean ± standard deviation. Statistical comparisons between two and multiple groups were performed using the unpaired Student’s t-test and one-way analysis of variance, respectively. All statistical analyzes were performed using GraphPad Prism version 9.5.1 (GraphPad, CA, USA), and *P* < 0.05 was considered statistically significant.

## Results

### Acute bowel obstruction adversely affects colonic anastomoses healing and induces gut dysbiosis in rat models

To establish an unfavorable healing environment, we developed an acute bowel obstruction model and examined its effects on colonic anastomotic healing in rat models. After 24 h of observation, faecal output ceased in the obstruction group, while the sham obstruction group maintained normal bowel function, followed by CPA, and there was no significant difference in the number of sutures (Fig. S1F). All experimental rats survived. ACSs was used to assess colonic anastomotic healing macroscopically. On POD 4, the obstruction group had a significantly higher score than the sham obstruction group (3.875 ± 0.991 vs. 2.250 ± 0.886, *P* < 0.01), indicating poorer healing (Fig. 1C). However, on POD 7, there was no significant difference in ACSs between the two groups (Fig. 1D).

Additionally, colonic anastomoses resilience was assessed via bursting pressure measurement, with no significant difference observed between the obstruction and sham obstruction groups on either POD 4 or 7 (Fig. 1E). Notably, on POD 7, most anastomoses in both groups withstood relatively high pressures (≥ 20 KPa), highlighting the strength achieved after seven days of healing [22]. Both groups exhibited a similar pattern of weight loss following initial surgery, with a further decrease observed on POD 4 and subsequent return to baseline levels by POD 7 (Fig. 1F).

Preoperative interventions (e.g., dietary regimens) drive gut microbiota remodeling, with specific taxa like *Bacteroides* showing expansion. Such dysbiotic patterns can adversely regulate colonic anastomosis repair processes [23]. To investigate whether the acute bowel obstruction model similarly leads to gut microbiota dysbiosis, which could be implicated in the pathological process of impaired colonic anastomotic healing, we conducted 16S rRNA sequencing analysis on fecal and mucosal samples obtained from both the obstruction and sham obstruction groups. Alpha analysis showed a significant reduction in mucosal microbial diversity in the obstruction group compared to the sham group (Fig. S3A), while no notable differences were found in fecal microbial diversity between the two groups (Fig. S3B). Principal coordinate analysis further demonstrated pronounced dissimilarities in mucosal microbiota composition between the two groups (Fig. S3C). Specifically, there was a marked increase in the abundance of *Bacteroides vulgatus* (*B. vulgatus*), while the abundance of *Lactobacillus intestinalis* (*L. intestinalis*) significantly decreased in the obstruction group (Fig. S3D).

### MSC-sEVs administration enhances colonic anastomoses healing in rat models

The adipose-derived MSCs were characterized (Fig. S4). MSC-sEVs characterisation was conducted as follows: First, sEVs morphology was examined using transmission electron microscopy (Fig. S5A). Nanoparticle tracking analysis revealed a peak vesicle size of approximately 100 nm (Fig. S5B). Additionally, western blotting was performed to detect marker proteins, including CD63, Alix, TSG101, and CD81 (Fig. S5C). PKH26-labeled MSC-sEVs were identified at the anastomotic site 12 h post-administration, with fluorescent signals primarily localized around the cell nuclei (Fig. S6), indicating effective cellular uptake.

On POD 4, we observed significantly better colonic anastomoses healing in the MSC-sEVs group, as indicated by a notably lower ACSs compared to the PBS group (2.375 ± 0.9161 vs. 3.750 ± 0.8864, *P* < 0.01) (Fig. 1G). Although the average bursting pressure was high in the MSC-sEVs group, the average bursting pressure was not significantly different between the two groups (Fig. 1H). In addition, weight changes were not significantly different between the two groups (Fig. 1I), and the suture number was comparable between groups(Fig. S1F).

**Fig. 2:**
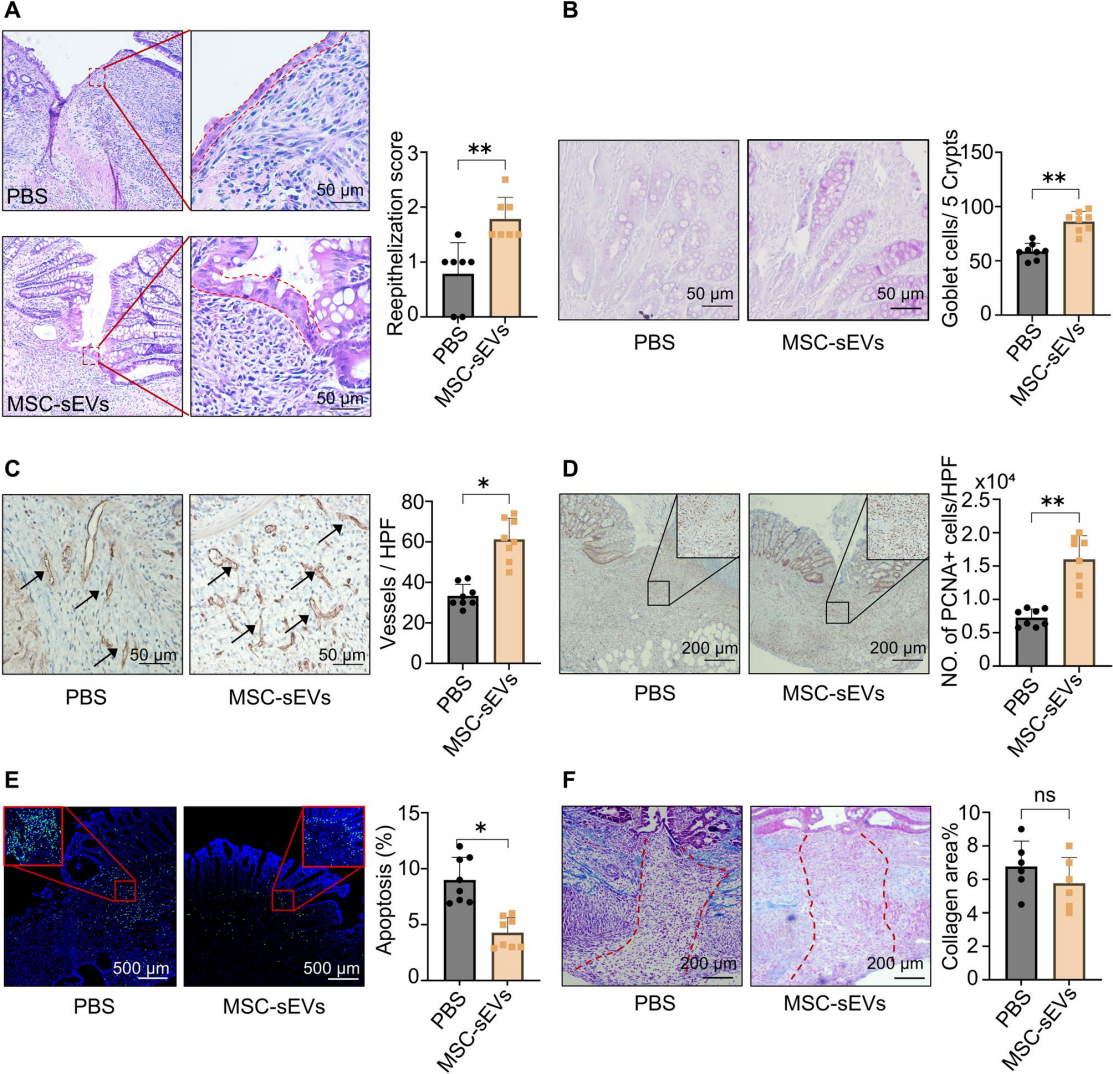
The histological assessment of colonic anastomoses. (**A**) Haematoxylin and Eosin staining shows re-epithelialization of the anastomoses. The red lines show the epithelium. (**B**) Periodic acid-Schiff staining shows the goblet cells. (**C**) CD31 immunohistochemistry staining. The black arrows indicate the blood vessels. (**D**) Immunohistochemistry staining of Proliferating Cell Nuclear Antigen (PCNA) shows active proliferative cells in the tissue. (**E**) The TUNEL (Terminal deoxynucleotidyl transferase dUTP nick end labeling) assay shows cells undergoing apoptosis in the tissue. (**F**) Masson’s trichrome staining. The blue area represents collagens, and the red lines highlight the region where collagens were absent. All data are shown as means ± SD. * *P* < 0.05. * * *P* < 0.01

Histological results demonstrated that epithelial repair in the colonic anastomoses was significantly better in the MSC-sEVs group compared to the PBS group (Fig. 2A), as indicated by the higher re-epithelialization score (Table S4). Moreover, in the MSC-sEVs group, a higher proportion of goblet cells, responsible for secreting mucin and maintaining the integrity of the intestinal mucosal barrier [24], was observed in the crypts surrounding the anastomoses (Fig. 2B). CD31 immunohistochemistry revealed a significantly greater density of blood vessels in the submucosal layer of the anastomosis site in the MSC-sEVs group compared to the control group (Fig. 2C). The development of new blood vessels around the anastomoses is essential for the supply of nutrients necessary for tissue repair. Additionally, a greater number of proliferating cells were observed in the submucosal layer treated with MSC-sEVs, whereas apoptotic cells were more abundant in the PBS-treated group (Fig. 2D E). Coherently, the microscopic enhancements in mucosal repair (re-epithelialization, goblet cells), angiogenesis, and cell turnover align with the superior macroscopic anastomotic integrity in the MSC-sEVs group.

Collagen depositions are important for anastomotic healing, and its impact on colonic anastomoses has been previously demonstrated [25]. Our study showed a clear lack of collagen deposition in colonic anastomoses on POD 4 in both PBS and MSC-sEVs groups, as demonstrated by Masson’s trichrome staining. However, further quantitative assessment of the collagen area ratio around the anastomosis showed no significant differences between the two groups (Fig. 2F). Additionally, the concentration of hydroxyproline in the colonic anastomotic tissue, a biochemical marker of collagen content, did not differ between the two groups (Fig. S7).

### MSC-sEVs effectively reduce inflammatory status in colonic anastomoses

**Fig. 3:**
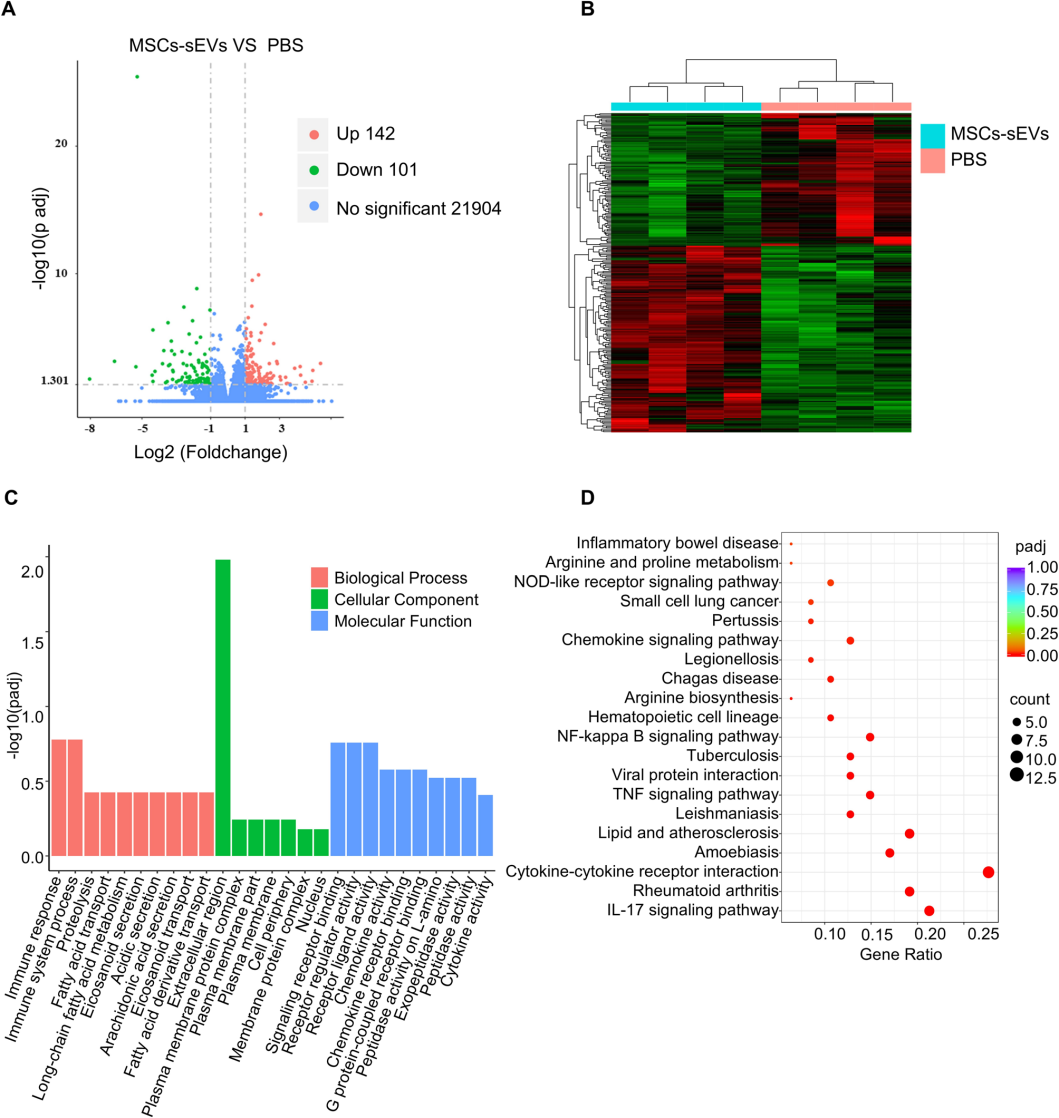
The gene expression analysis of colonic anastomoses. (**A**, **B**) Volcano plots and clustering heatmap of anastomotic tissue mRNA sequencing. (**C**, **D**) Gene Ontology and Kyoto Encyclopedia of Genes and Genomes analysis of mRNA sequencing results

To investigate gene expression differences in colonic anastomotic tissues between the MSC-sEVs and PBS groups, we conducted whole-transcriptome sequencing, and 142 genes were significantly upregulated, whereas 101 were considerably downregulated in the MSC-sEVs group (Fig. 3A). The hierarchical clustering analysis revealed distinct expression patterns of DEGs between the two groups (Fig. 3B). GO analysis indicated that these DEGs were primarily associated with immune response, extracellular matrix regulation, and intercellular communication (Fig. 3C). Importantly, KEGG enrichment analysis highlighted key pathways (Fig. 3D), indicating the downregulation of immune and inflammatory pathways, including the nuclear factor (NF)-κB, interleukin (IL)-17, and tumor necrosis factor (TNF) signaling pathways in the MSC-sEVs group. Activation of these three pathways serves as key mediators of pro-inflammatory responses, capable of promoting immune cell recruitment and enhancing the expression of various pro-inflammatory cytokines and chemokines [26–28].

**Fig. 4:**
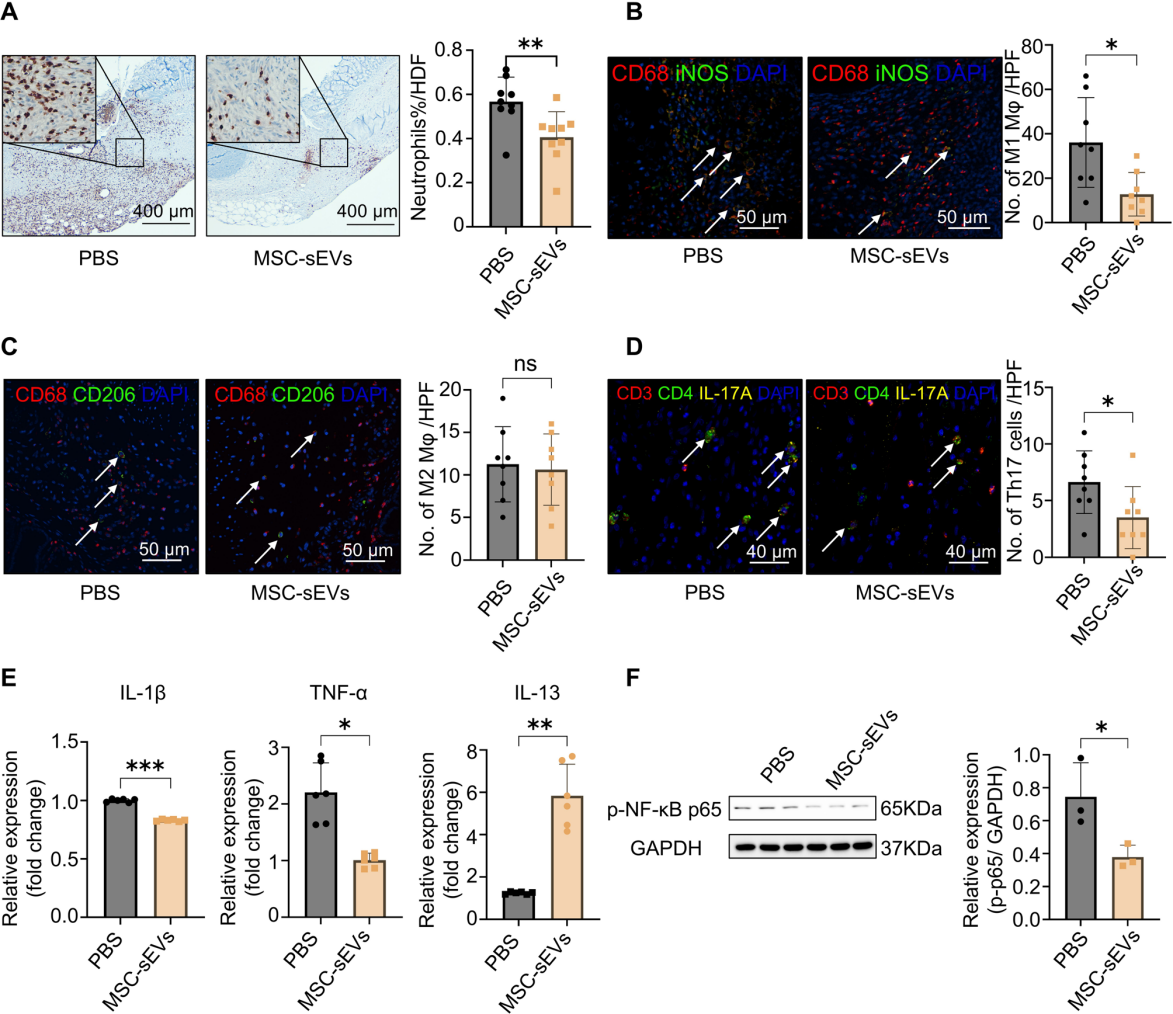
The inflammation analysis of the colonic anastomoses. (**A**) Myeloperoxidase (MPO) immunohistochemistry staining shows neutrophils in the anastomotic tissue. (**B**) Double immunofluorescence staining of CD68 (red) and iNOS (green) shows M1 macrophages (the white arrows indicate). (**C**) Double immunofluorescence staining of CD68 (red) and CD206 (green) shows M2 macrophages (the white arrows indicate). (**D**) Polychromatic immunofluorescent staining of CD3 (red), CD4 (green) and IL-17 A (yellow) shows Th17 cells (the white arrows indicate). (**E**) Expression of the cytokines (interleukin (IL)-1β, tumour necrosis factor (TNF)-α and IL-13) in the anastomotic tissue was detected via real-time polymerase chain reaction. (**F**) Western blot of phospho(p)-NF-κB p65 expression in the colon anastomotic tissue. Full-length blots are presented in Supplementary Fig. 10. All data are shown as means ± SD. * *P* < 0.05. * * *P* < 0.01

To critically assess acute inflammation in colonic anastomosis tissue, we examined neutrophil infiltration with a myeloperoxidase marker [29]. The results indicated that neutrophil infiltration was significantly higher in the PBS group than in the MSC-sEVs group (Fig. 4A). Macrophages are critical immune cells that play a significant role in immune response and tissue repair, often recruited to the injury site after neutrophils [30]. We assessed the expression of the macrophage subtypes in the tissue. M1 macrophages, known for their pro-inflammatory role, were significantly less prevalent in the MSC-sEVs group compared to the PBS group (Fig. 4B & Fig. S8). However, M2 macrophages, recognized for anti-inflammatory properties, exhibited no significant differences in their expression between the two groups (Fig. 4C). Th17 cells are key producers of IL-17 cytokines, and IL-17 plays a central role in activating the IL-17 signaling pathway [31]. Among the IL-17 cytokine family, IL-17 A (hereafter referred to as IL-17 unless otherwise specified) is the most extensively studied and representative member, with well-established roles in the pathogenesis of various inflammatory disorders [28, 32]. To investigate the involvement of Th17 cells in our model, we characterised the infiltration of these cells in colonic tissues through polychromatic immunofluorescent staining for CD3, CD4, and IL-17 A [33]. Results revealed fewer Th17 cells in the colonic tissues of the MSC-sEVs group compared to the PBS control group (Fig. 4D).

Furthermore, we performed real-time reverse polymerase chain reaction (RT-qPCR) to examine the transcriptional expression of key genes, demonstrating that the expression levels of pro-inflammatory cytokines IL-1β and TNF-α significantly decreased, whereas the levels of the anti-inflammatory mediator IL-13 significantly increased in the anastomotic tissues of the MSC-sEVs group compared to the PBS group (Fig. 4E). At the protein level, western blotting analysis showed that phosphorylation of NF-κB p65, which is crucial for NF-κB pathway activation, was reduced in the MSC-sEVs group (Fig. 4F).

### MSC-sEVs inhibit LPS-induced M1 macrophage polarisation and NF-κB pathway activation in vitro

**Fig. 5:**
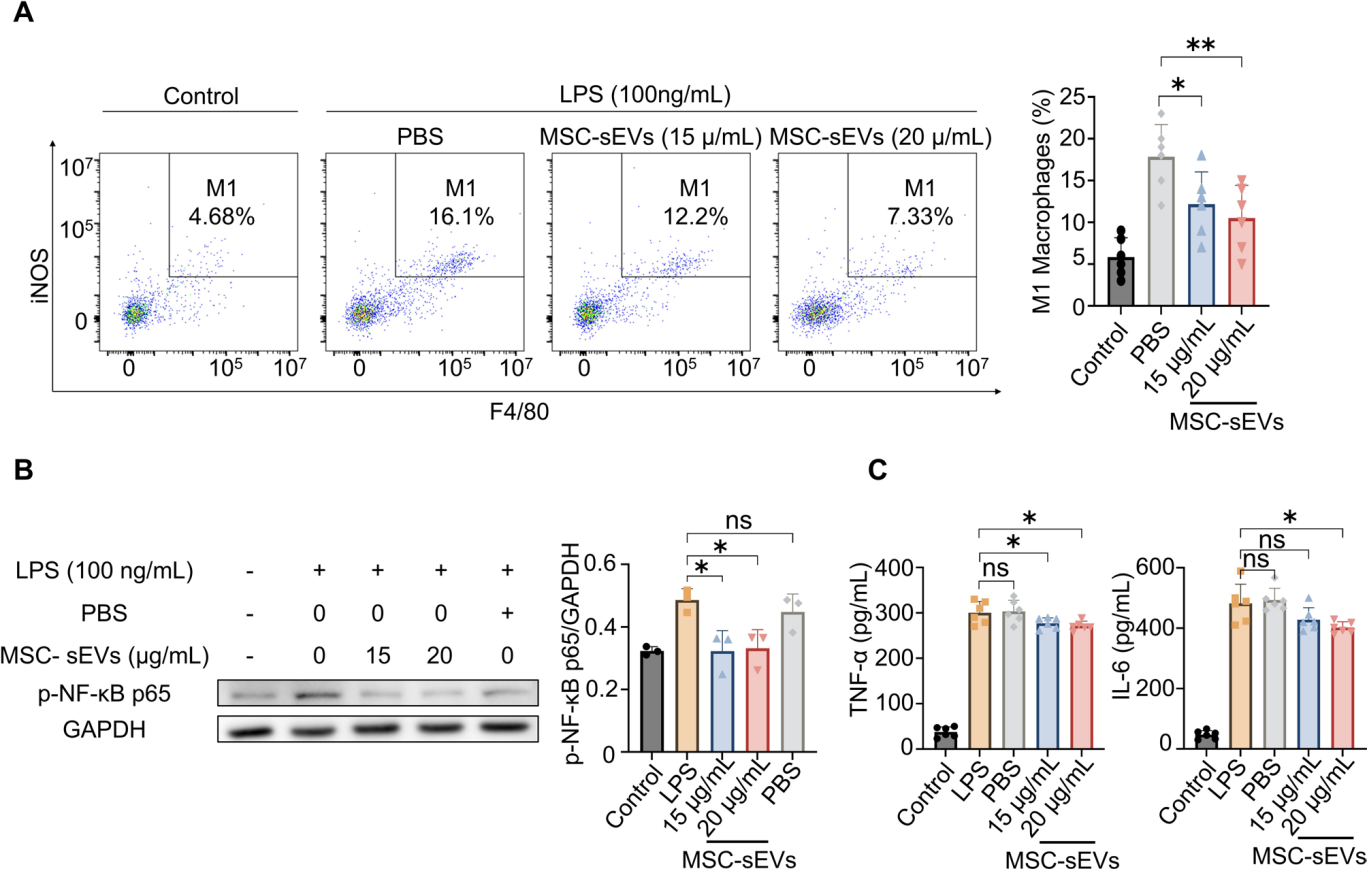
The experiments in vitro. (**A**) Flow cytometry analysis of M1 macrophage ratio of RAW 264.7 cells. (**B**) Western blot of phospho-NF-κB p65 expression in RAW 264.7 cells. Full-length blots are presented in Supplementary Fig. 11. (**C**) Enzyme-linked immunosorbent assay analysis (ELISA) of TNF-α and IL-6 in the RAW 264.7 cell culture supernatant. All data are shown as means ± SD. * *P* < 0.05. * * *P* < 0.01

The RAW 264.7 cell line was used to validate the anti-inflammatory effects of MSC-sEVs in vitro. Lipopolysaccharide (LPS), a component of bacterial cell walls, can elicit an inflammatory response in macrophages, driving their polarization toward the M1 phenotype [34]. LPS was added to the culture medium to induce an inflammatory response in the RAW 264.7 cells before treatment with MSC-sEVs or PBS. MSC-sEVs treatment attenuated the inflammatory response in LPS-activated macrophages: the percentage of M1 macrophages was significantly lower in the MSC-sEVs group than in the PBS group (Fig. 5A). Additionally, phosphorylation of NF-κB p65 was significantly lower in the MSC-sEVs group, while no notable changes were observed in the PBS group (Fig. 5B). Furthermore, levels of the pro-inflammatory cytokines, TNF-α and IL-6, in the cell culture supernatant decreased after MSC-sEVs treatment (Fig. 5C).

As supplementary observations, we found that MSC-sEVs also enhanced the proliferative and migratory capabilities of CCD 841 CoN cells (Fig. S9A B) and promoted angiogenesis in HUVECs (Fig. S9C) in vitro. The MSC-sEVs were taken up into the cytoplasm by the cells in vitro after co-culture for six hours (Fig. S9D).

## Discussion

In clinical practice, AL is diagnosed through complementary assessments, such as faecal drainage observation and CT scans [35]. However, these methods are challenging to implement in animal experiments. According to an international consensus on intestinal anastomosis models, employing an appropriate scoring system facilitates a more objective and accurate evaluation of anastomotic healing compared to the conventional AL rate [20]. Moreover, it is important to note that rodents generally exhibit a stronger healing capacity than humans, which may lead to less pronounced differences in research outcomes [36]. This explains the preliminary experimental results indicating that although there was markedly poor anastomotic healing on POD 4 due to acute bowel obstruction, healing occurred by POD 7, a phenomenon rarely observed in clinical practice.

As previously mentioned, the healing of colonic anastomoses generally follows three main stages: inflammatory, proliferative, and remodeling phases [7]. It is important to note that in a typical healing process, the inflammatory phase is largely completed by POD 4, transitioning into the proliferative phase. However, adverse factors including ischemia, edema, and various injuries, can prolong activation of inflammation, resulting in tissue damage and impeding the overall healing process [37]. In this study, acute bowel obstruction, classified as a physical injury, was found to adversely affect anastomotic healing during the early postoperative stage (POD4). Additionally, we also observed that dysbiosis of the colon mucosal gut microbiota in this acute bowel obstruction model was characterised by a significant increase in *B. vulgatus*, accompanied by a marked decrease in *L. intestinalis*. Although *B. vulgatus* is normally a commensal organism in the human gut, its abnormal proliferation under specific conditions can precipitate colonic inflammation and has been associated with the clinical onset of ulcerative colitis [38]. Conversely, *L. intestinalis* are crucial for maintaining the integrity of the intestinal mucosal barrier [39]. Consequently, this dysbiosis pattern may result in compromised barrier function, thereby exacerbating the obstructed colon associated injuries and inflammation. Interestingly, these alterations were observed only in the mucosal microbiota, with no differences noted in the faecal microbiota. This suggests that the mucosal microbiota is more reflective of disease states, aligning with findings from previous clinical studies [40].

MSCs, a multipotent subset of adult stem cells, are derived from diverse tissue sources such as adipose tissue, bone marrow, and umbilical cord, among others [41]. While MSCs derived from different sources exhibit some differences in their differentiation capabilities, their immunoregulatory functions remain largely comparable [42, 43]. Remarkably, adipose tissue offers several advantages: it yields a greater number of stem cells with enhanced viability, and the extraction process is less invasive, making it easier to obtain through procedures such as liposuction [41]. These characteristics indicate that adipose-derived MSCs possess substantial potential for clinical translation.

The role of MSC- sEVs in regulating immunomodulatory inflammation has been widely established [44–47]. In this study, topical administration of MSC-sEVs effectively mitigated the rat colon anastomotic impairment by suppressing excessive inflammatory responses on POD4, as evidenced by diminished neutrophil infiltration, reduced pro-inflammatory M1 macrophages and Th17 cells, along with downregulation of pro-inflammatory pathways including NF-κB, IL-17, and TNF pathways. Besides, the release of pro-inflammatory cytokines, including TNF-α and IL-1β, decreased. A key mechanistic insight lies in the dual-functional regulation of M1 macrophages. Although these cells generate antimicrobial reactive species such as nitric oxide and superoxide to eliminate pathogens, their uncontrolled activity drives indiscriminate tissue cytotoxicity, exacerbating anastomotic damage [48]. Our findings reveal that MSC-sEVs normalized M1 macrophage hyperactivity by POD4, effectively mitigating their detrimental effects on anastomotic healing. Similarly, IL-17 exhibits a dichotomous role in wound healing: transient signaling facilitates host defense, whereas persistent activation induces destructive immunopathology and impedes regeneration [32, 49]. Notably,​​ the observed suppression of Th17 cell infiltration in MSC-sEVs-treated groups mirrors this balance, demonstrating​ a therapeutic mechanism that prevents prolonged Th17-mediated dysregulation. Furthermore, IL-17 can also recruit neutrophils and activate the NF-κB pathway, establishing a self-reinforcing inflammatory loop that affects macrophage infiltration and polarization [28, 50]. Collectively, the coordinated attenuation of M1 macrophage hyperactivity and Th17-mediated inflammation by MSC-sEVs underscores their multimodal therapeutic potential in optimizing the inflammatory microenvironment for wound repair. Nevertheless, the crosstalk between macrophages and Th17 cells in this regulatory network requires further elucidation​ to optimize MSC-sEVs-based strategies for colonic anastomotic repair [51].

At the in vitro level, we observed that under the same LPS stimulation conditions, co-culturing with MSC-sEVs significantly reduced the proportion of M1 macrophages, while activation of the NF-κB pathway was inhibited, and the levels of pro-inflammatory factors TNF-α and IL-6 in the supernatant were similarly suppressed, corroborating the anti-inflammatory effect of MSC-sEVs across models. Additionally, we observed the pro-regenerative effects of MSC-sEVs in vitro assessments, including increased proliferation and migration of CCD-841-CoN cells and enhanced HUVEC tube formation. These findings are consistent with previous studies indicating that MSC-sEVs deliver Wnt agonists (e.g., Wnt4) and regulatory microRNAs (e.g., miR-136-5p), which activate the Wnt/β-catenin pathway and subsequently trigger downstream targets to promote epithelial cell cycling [52–54]. Furthermore, the enhancement of HUVEC tube formation by MSC-sEVs involves PTEN/AKT pathway regulation; this effect is also associated with microRNAs contained within MSC-sEVs, such as miR-135b-5p and miR-499a-3p [55, 56]. Undoubtedly, the role of MSC-sEVs in enhancing cell proliferation and angiogenesis is crucial for promoting wound healing [57, 58]. ​ Given that our transcriptomic data primarily highlighted differential regulation of immune-inflammatory pathways, we propose that the immunomodulatory effects of MSC-sEVs in the early stages are particularly critical for improving colonic anastomotic healing. By attenuating excessive immune activation, these effects may establish a foundation for subsequent resident cell proliferation, epithelial repair, and neovascularization. This aligns with the widely recognized wound healing paradigm, wherein resolution of inflammation is a prerequisite for the transition to proliferative phases [37, 59]. Clinically, the temporal association between colonic AL incidence and early postoperative inflammation further emphasizes​ the critical window for MSC-sEVs-mediated immunomodulation to secure healing outcomes [9].

Previous studies demonstrated that collagen deposition significantly influences intestinal AL rate [25, 60]. However, we did not observe a significant effect of MSC-sEVs on collagen deposition. This may be due to the relatively early time point of our outcome assessment (POD 4), during which collagen fiber degradation is predominant, and collagen levels are likely to increase until POD 7 [61]. Thus, the early phase of colonic anastomotic healing facilitated by MSC-sEVs may not be strongly associated with collagen fiber repair.

This study has several limitations. Although we found that gut dysbiosis was involved in the impaired anastomotic healing caused by acute bowel obstruction, further research is necessary to elucidate the specific mechanisms [23]. Additionally, rat experiments exhibit notable limitations owing to interspecies differences and the established model’s inability to accurately replicate clinical settings. Nonetheless, the promising preclinical results offer valuable insights and technical support for further clinical trials. While MSC-sEVs safety continues to be debated, emerging clinical evidence supports their tolerability and therapeutic potential in tissue repair, warranting further efficacy and safety validation [62–64].

## Conclusions

In conclusion, acute bowel obstruction compromises the healing of colonic anastomoses. This study is the first to demonstrate the application of MSC-sEVs in a colonic anastomosis animal model, under conditions of bowel obstruction. Our findings elucidate the therapeutic role of MSC-sEVs in anastomotic healing, primarily through the attenuation of excessive inflammation during the early healing phases of tissue repair. These promising preclinical results provide new insights and evidence for developing strategies aimed at improving colonic anastomosis healing in clinical settings.

## Supplementary Information

Below is the link to the electronic supplementary material.

Supplementary Material 1

Supplementary Material 2

Supplementary Material 3

## Data Availability

All data generated or analysed during this study are included in this published article and its supplementary information files.

## Abbreviations

MSCs: Mesenchymal stem cells
MSCs-sEVs: MSCs derived small EVs
AL: Anastomotic leakage
ACSs: Anastomotic complication scoring system
LPS: Lipopolysaccharide
TNF-α: Tumor necrosis factorα
IL: Interleukin
iNOS: Inducible nitric oxide synthase
PBS: Phosphate-buffered saline
FBS: Fetal bovine serum
H&E: Hematoxylin and eosin
DAPI: 4’0.6-diamidino-2-phenylindole
PAS: Periodic acid-schiff
ELISA: Enzyme-linked immunosorbent assay
SDS-PAGE: Sodium dodecyl sulfate-polyacrylamide gel electrophoresis
PVDF: Polyvinylidene difluoride
GAPDH: Glyceraldehyde-3-phosphate dehydrogenase
HRP: Horseradish peroxidase-conjugated
PCNA: Proliferating cell nuclear antigen
CCK-8: Cell counting kit 8
MPO: Myeloperoxidase
DAMPs: Direct damage-associated molecular patterns
PAMPs: Pathogen-associated molecular patterns
