# Supplementary material for: Mesenchymal stem cell-derived small extracellular vesicles facilitate repair of acute obstruction-induced colonic anastomosis injury by modulating early-stage inflammation in rats
Source: Stem Cell Res Ther. 2025 Aug 6;16:433. doi: 10.1186/s13287-025-04551-8 (PMC12329984; doi:10.1186/s13287-025-04551-8)
Supplement: Supplementary file 1 — Supplementary Material 1 [file 13287_2025_4551_MOESM1_ESM.pdf]

Supplementary Material 1

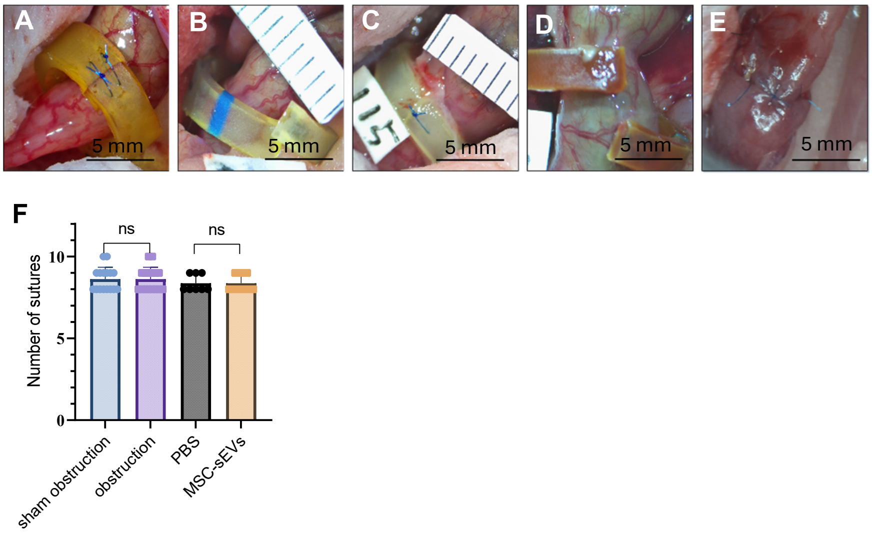

Fig. S1 (A) Colon obstruction operation using a silicone ring. (B) The sham obstruction procedure. (C D) Colon condition 24 h after colon obstruction or sham obstruction. (E) The end-to-end anastomosis. (F) Number of sutures in different groups. All data are shown as means ± SD.

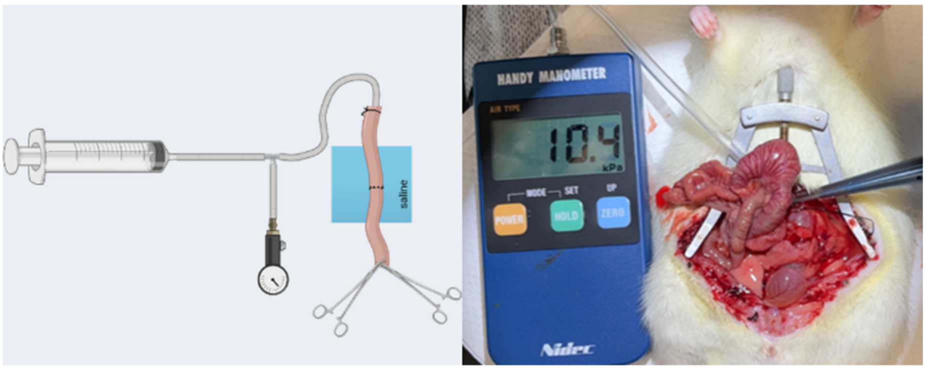

Fig. S2 Anastomotic bursting pressure test in situ.

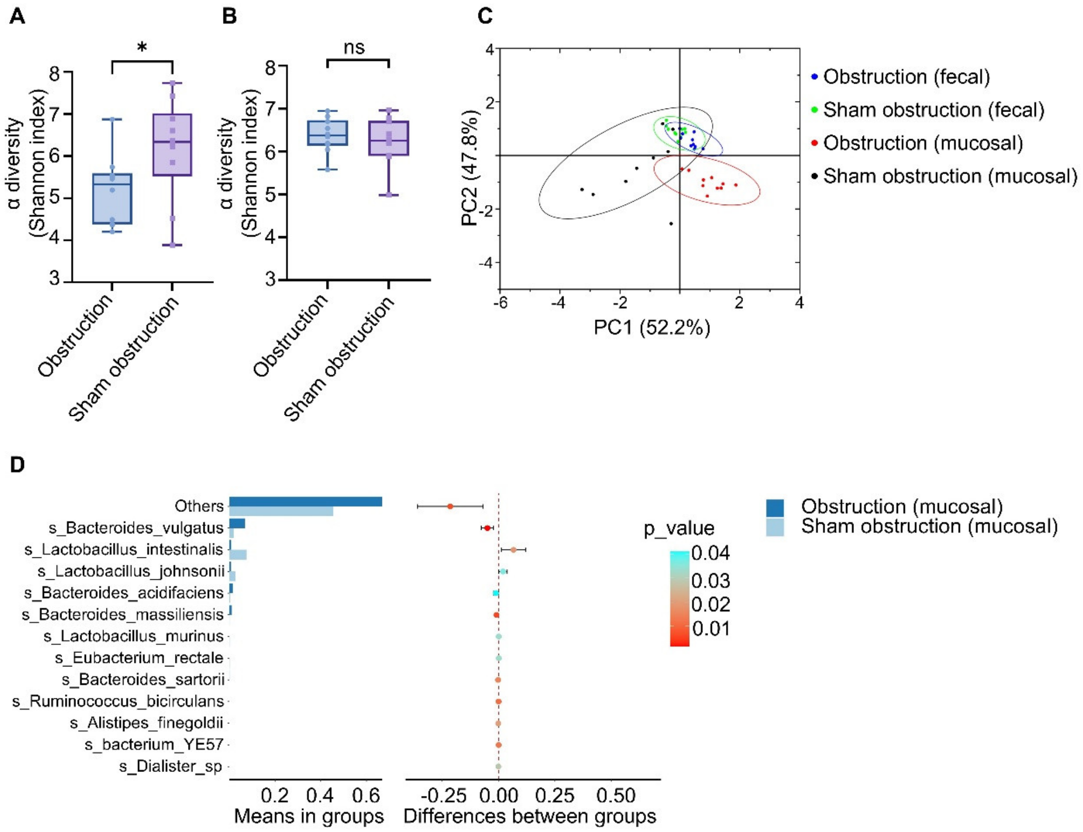

Fig. S3 16S ribosomal RNA sequencing analysis. (A) The alpha analysis of mucosal microbial community (n = 10). (B) Alpha analysis of faecal microbial community (n = 10). (C) Principal coordinate analysis of mucosal microbial community (n = 10). (D) A student-test was performed to determine species of mucosal microbiota with significant variation between groups (P < 0.05). The left panel shows the abundance of species showing significant differences between groups; the right panel represents the confidential interval of between-group variations (n = 10). All data are shown as means ± SD. * P < 0.05.

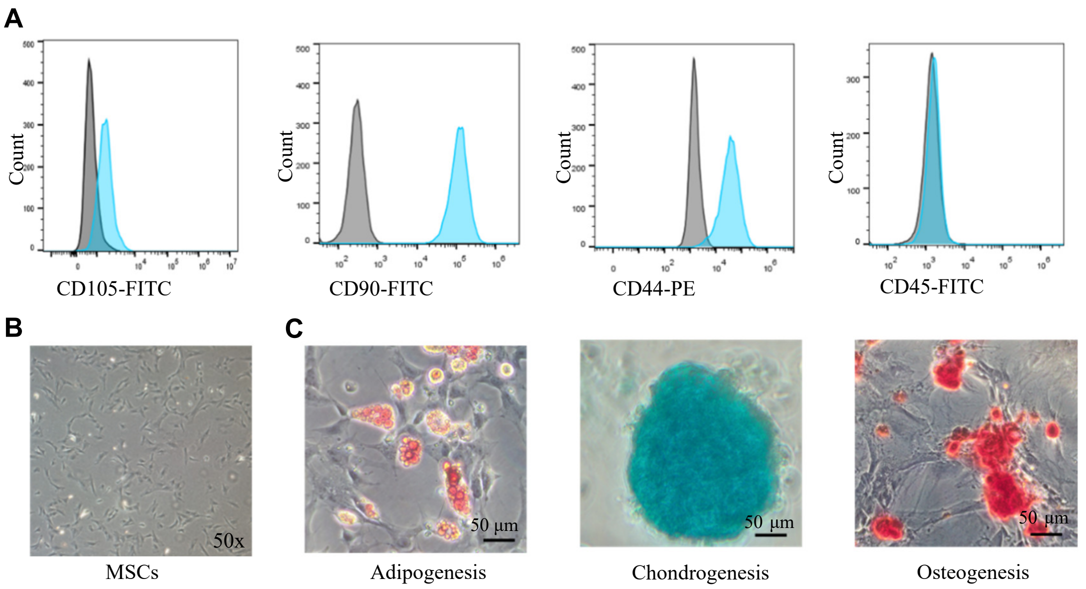

Fig. S4 (A) The flow cytometry identifies positive marker proteins (CD29, CD90, CD105, CD44) and negative protein (CD45). (B) The spindle morphology of MSCs. (C) The differentiation potential of MSCs for adipogenesis, chondrogenesis, and osteogenesis.

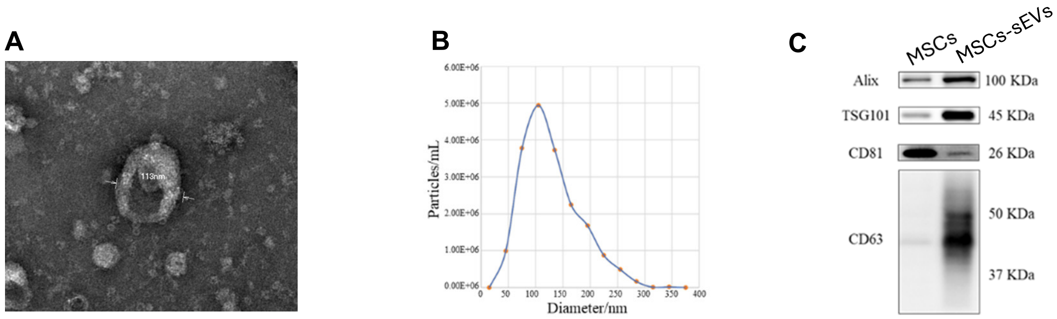

Fig. S5 (A) The transmission electron microscopy of MSC-sEVs. (B) Nanoparticle Tracking Analysis of MSC-sEVs. (C) The western blot analysis of MSC-sEVs for positive proteins CD63, Alix, CD81, and Tsg101. Full-length blots/gels are presented in Supplementary Figure 12.

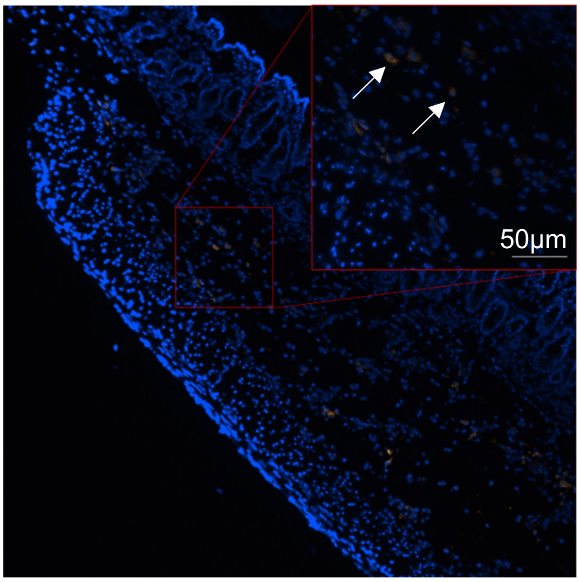

Fig. S6 PKH26-labeled MSC-sEVs were tracked in vivo. The white arrows indicate the MSC-sEVs around the cell nuclei.

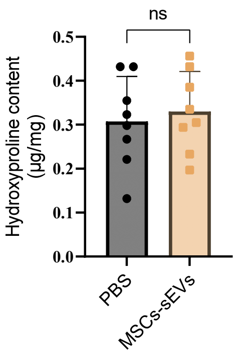

Fig. S7 Hydroxyproline concentration analysis of the anastomose tissue on POD 4. All data are shown as means ± SD. * P < 0.05. * * P < 0.01.

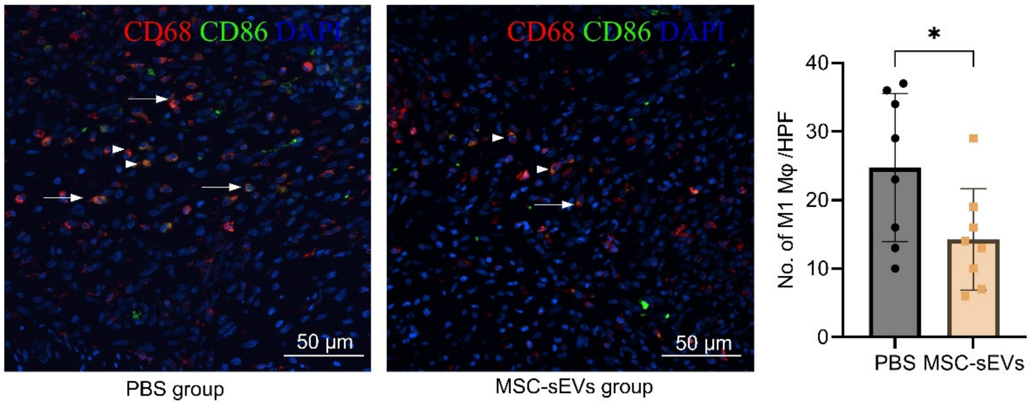

Fig. S8 Double immunofluorescence staining of CD68 (red) and CD86 (green) shows M1 macrophages (the white arrows indicate). All data are shown as means ± SD. * P < 0.05.

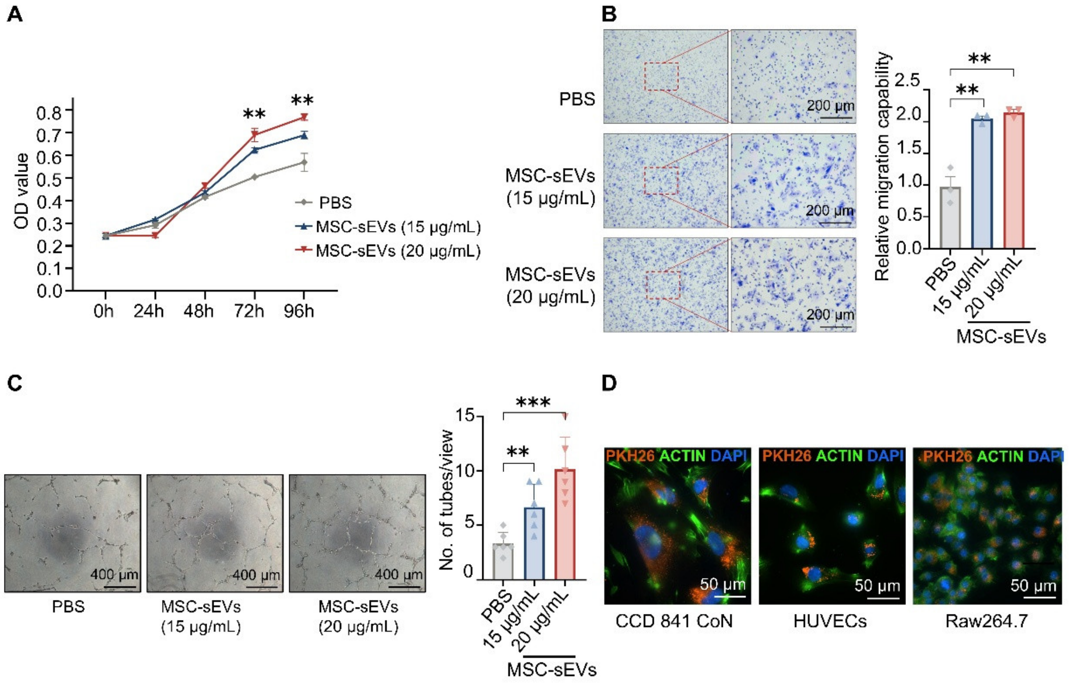

Fig. S9 . (A B) Cell Counting Kit-8 (CCK8) assay and the transwell migration assay of CCD 841 CoN cells treated with phosphate-buffered saline (PBS) or MSC-sEVs. (C) Tube formation assay of human umbilical vein endothelial cells (HUVECs). (G) The immunofluorescence staining shows the PKH26 labelled MSC-sEVs were taken up into the cytoplasm. All data are shown as means ± SD. * * P < 0.01. * ** P < 0.001.

Table S1. Anastomotic complication score system.

Levels	Scores	Items
I	0	No adhesion or abnormalities;
IIa	1	Light adhesion to fat pad; clean anastomosis underneath adhesions;
IIb	2	Dense adhesion to intestinal loop, abdominal wall, or another organ; clean anastomosis underneath adhesions;
IIIa	3	Signs of localized contamination - one small abscess (≤ 2 mm);
IIIb	4	Signs of localized contamination - one big abscess (> 2 mm) or more than one abscess;
IIIc	5	Signs of localized faecal contamination or obvious defects;
IV	6	Signs of general contamination - spread of pus, faeces; ascites or peritonitis;

The anastomotic healing condition was assessed macroscopically using the anastomotic complication score system, which was modified based on previous studies[30].

Table S2. Sequences of primers used in real-time polymerase chain reaction (PCR).

**Primer**	**Sequences (5′→3′)**
IL-1β-F	AGGAGAGACAAGCAACGACA
IL-1β-R	TTTGGGATCCACACTCTCCAG
TNF-α-F	ATGGGCTCCCTCTCATCAGT
TNF-α-R	GCTTGGTGGTTTGCTACGAC
IL-13-F	ATCACACAAGACCAGAAGACTTC
IL-13-R	AACTGGGCTACTTCGATTTTGG
GAPDH-F	GGCACAGTCAAGGCTGAGAATG
GAPDH-R	ATGGTGGTGAAGACGCCAGTA

IL, interleukin; TNF: tumor necrosis factor, GAPDH: glyceraldehyde-3-phosphate

Table S3. Antibodies used in western blot assay.

**Antibodies**	**Company**	**Catalog No.**
GAPDH	Cell Signaling Technology	5174
Alix	Abcam	ab117600
TSG101	Abcam	Ab125011
CD63	Invitrogen	MA5-35208
CD81	Abcam	ab109201
PCNA	Abcam	ab152112
Phospho-NF-κB p65	Cell Signaling Technology	3033

GAPDH, glyceraldehyde-3-phosphate; TSG, Tumor Susceptibility Gene; PCNA, Proliferating Cell Nuclear Antigen.

Table S4. Anastomotic re-epithelialization score system.

Levels	Scores	Items
I	0	No epithelization
II	1	Incomplete coverage
III	2	Complete coverage (single cell layer)
IV	3	Complete re-epithelization (glandular epithelium)
