# Supplementary figures and images for: Mesenchymal stem cell-derived small extracellular vesicles facilitate repair of acute obstruction-induced colonic anastomosis injury by modulating early-stage inflammation in rats
Source: Stem Cell Res Ther. 2025 Aug 6;16:433. doi: 10.1186/s13287-025-04551-8 (PMC12329984; doi:10.1186/s13287-025-04551-8)

Supplementary Figure 10

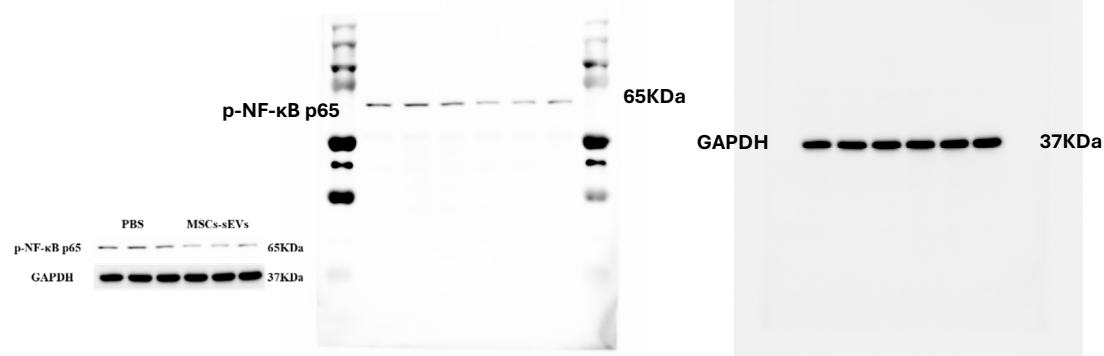

Supplementary Figure 11

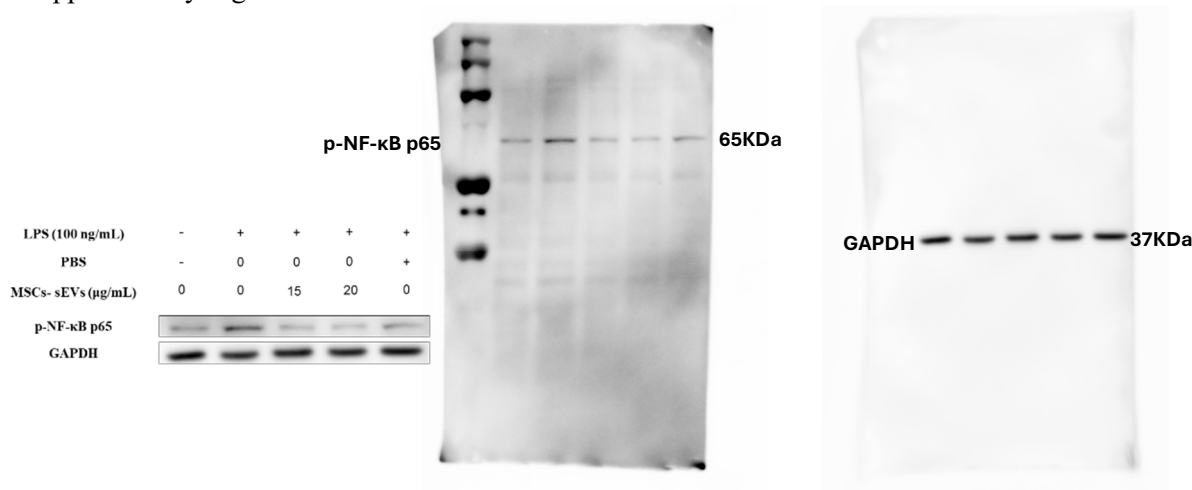

Supplementary Figure 12

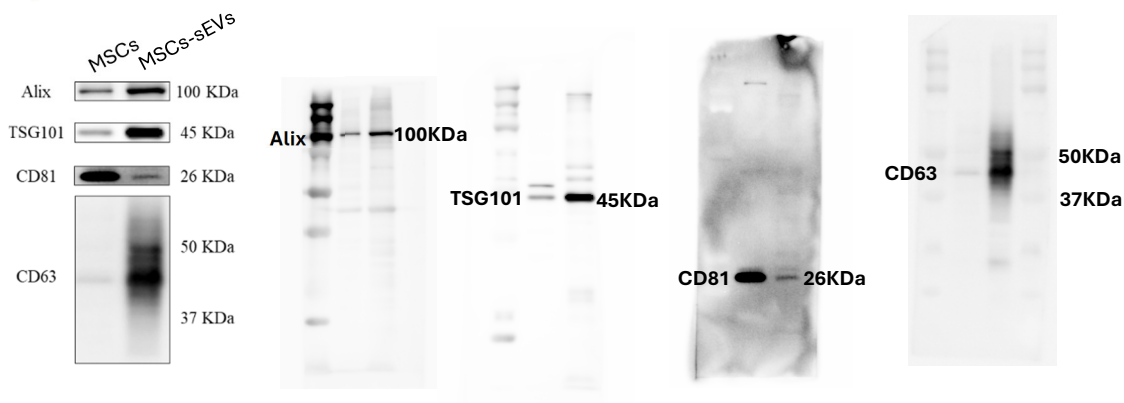

Supplement: Supplementary file 2 — Supplementary Material 2 [file 13287_2025_4551_MOESM2_ESM.pdf]
